# *CEBPE*-Mutant Specific Granule Deficiency Correlates With Aberrant Granule Organization and Substantial Proteome Alterations in Neutrophils

**DOI:** 10.3389/fimmu.2018.00588

**Published:** 2018-03-29

**Authors:** Nina K. Serwas, Jakob Huemer, Régis Dieckmann, Ester Mejstrikova, Wojciech Garncarz, Jiri Litzman, Birgit Hoeger, Ondrej Zapletal, Ales Janda, Keiryn L. Bennett, Renate Kain, Dontscho Kerjaschky, Kaan Boztug

**Affiliations:** ^1^Ludwig Boltzmann Institute for Rare and Undiagnosed Diseases, Vienna, Austria; ^2^CeMM Research Center for Molecular Medicine of the Austrian Academy of Sciences, Vienna, Austria; ^3^Clinical Institute of Pathology, Medical University of Vienna, Vienna, Austria; ^4^Department of Pediatric Hematology and Oncology, 2nd Faculty of Medicine, University Hospital Motol, Prague, Czechia; ^5^Department of Clinical Immunology and Allergology, St. Anne’s University Hospital, Faculty of Medicine, Masaryk University, Brno, Czechia; ^6^Department of Pediatric Hematology, University Hospital Brno, Brno, Czechia; ^7^Center for Chronic Immunodeficiency (CCI), University Medical Center, University of Freiburg, Freiburg, Germany; ^8^Center of Pediatrics and Adolescent Medicine, University Medical Center, University of Freiburg, Freiburg, Germany; ^9^Department of Pediatrics and Adolescent Medicine, Medical University of Vienna, Vienna, Austria; ^10^Department of Pediatrics, St. Anna Kinderspital and Children’s Cancer Research Institute, Medical University of Vienna, Vienna, Austria

**Keywords:** primary immunodeficiency, neutrophil granulocytes, granule organization, C/EBPε, specific granule deficiency

## Abstract

Specific granule deficiency (SGD) is a rare disorder characterized by abnormal neutrophils evidenced by reduced granules, absence of granule proteins, and atypical bilobed nuclei. Mutations in *CCAAT/enhancer-binding protein-ε* (*CEBPE*) are one molecular etiology of the disease. Although C/EBPε has been studied extensively, the impact of *CEBPE* mutations on neutrophil biology remains elusive. Here, we identified two SGD patients bearing a previously described heterozygous mutation (p.Val218Ala) in *CEBPE*. We took this rare opportunity to characterize SGD neutrophils in terms of granule distribution and protein content. Granules of patient neutrophils were clustered and polarized, suggesting that not only absence of specific granules but also defects affecting other granules contribute to the phenotype. Our analysis showed that remaining granules displayed mixed protein content and lacked several glycoepitopes. To further elucidate the impact of mutant *CEBPE*, we performed detailed proteomic analysis of SGD neutrophils. Beside an absence of several granule proteins in patient cells, we observed increased expression of members of the linker of nucleoskeleton and cytoskeleton complex (nesprin-2, vimentin, and lamin-B2), which control nuclear shape. This suggests that absence of these proteins in healthy individuals might be responsible for segmented shapes of neutrophilic nuclei. We further show that the heterozygous mutation p.Val218Ala in *CEBPE* causes SGD through prevention of nuclear localization of the protein product. In conclusion, we uncover that absence of nuclear C/EBPε impacts on spatiotemporal expression and subsequent distribution of several granule proteins and further on expression of proteins controlling nuclear shape.

## Introduction

Neutrophil granulocytes are the first line of defense that is recruited to sites of infection. Mature neutrophils contain a number of preformed receptors and microbicidal peptides stored in distinct subcellular organelles. The regulated fusion of these granules with their target membrane controls both extravasation of cells from the blood stream to inflammatory sites and the ability of cells to sense, ingest, and destroy microbes (1). The nomenclature of the classical granules reflects the order at which they are formed during myeloid differentiation in the bone marrow (2): primary (or azurophilic) granules characteristically contain myeloperoxidase (MPO) and appear at the promyelocyte stage, secondary granules are marked by their high content of lactoferrin and transcobalamin-1, and tertiary granules are defined by a high content in neutrophil gelatinase and appear after the myelocyte stage (3). Secretory vesicles are formed only very late in the maturation process and contain several membrane-associated molecules (CD16b and CD13) and are constituted of endocytosed material (4).

Quantitative or qualitative neutrophil deficiencies are associated with an often remarkable vulnerability to bacterial and fungal infections [reviewed in Ref. (5, 6)]. Among these defects, specific granule deficiency (SGD) denotes a particularly rare primary immunodeficiency with a handful of cases reported to date (7–11). Clinically, patients suffer from recurrent infections and abscesses in airways and skin. Diagnosis is based on the characteristic aspect of peripheral blood neutrophils, which are not fully differentiated but, instead, display atypically bilobed nuclei and abnormal granule numbers and content (7, 8, 11). One of these features, namely, hypogranularity, resembles myelodysplastic syndrome (12). The scarcity of published cases and lack of diagnostic criteria make the diagnosis challenging, suggesting that the true disease prevalence may be underestimated. In SGD, neutrophils lack the expression of granule proteins localized to specific and gelatinase granules or localized to azurophilic granules but expressed in the late promyelocyte stage, like bactericidal permeability-increasing protein (BPI) and defensins or human neutrophil peptides (HNPs) (13). Eosinophils have been also reported to contain abnormal granules (14). Neutrophils from patients with SGD are impaired in chemotaxis and in killing of *Staphylococcus aureus* bacteria *in vitro* (7, 13).

Mutations in *CEBPE*, encoding the transcription factor CCAAT/enhancer-binding protein-ε (C/EBPε), are one of the known genetic causes of SGD (9, 15–17). Of note, more recently another genetic etiology of SGD has been identified, caused by biallelic mutations in the gene *SMARCD2* encoding an interactor of C/EBPε (10). C/EBPε is a critical transcription factor for the normal differentiation of neutrophils beyond the promyelocytic stage in the bone marrow (12). Accordingly, it directly regulates the transcription of components of the secondary/tertiary granules in neutrophils and eosinophils (17–20) and indirectly regulates nuclear segmentation (21).

Interestingly, both homozygous and heterozygous mutations in *CEBPE* have been identified in SGD patients. Two homozygous mutations are frameshift mutations located in exon 1 of *CEBPE* leading to the expression of a truncated protein lacking transcriptional activity (15, 16). Another homozygous mutation results in an in-frame deletion of two amino acids inside the leucine zipper domain of the protein (9). By contrast, the observed SGD-causative heterozygous mutation is presumed to act as a dominant missense mutation by amino acid substitution from valine to alanine at position 218 (c.653C/T>C; p.Val218Ala). The altered amino acid is located in the basic leucine zipper (b-zip) domain of C/EBPε (17). The molecular details how this heterozygous point mutation in *CEBPE* leads to a similar phenotype as the homozygous frameshift mutation is only partially understood. In this study, we investigated the pathological mechanisms for disease onset in two patients bearing the C/EBPε^Val218Ala^ variant and performed detailed neutrophil granule and protein content analysis to gain novel molecular insights into the pathophysiology of SGD.

## Materials and Methods

### Patients and Control Individuals

This study was carried out in accordance with the recommendations of the institutional review boards of the Medical University of Vienna with written informed consent from all subjects. All subjects gave written informed consent in accordance with the Declaration of Helsinki. The protocol was approved by the institutional review boards of the Medical University of Vienna. Patient 1 was 3–9 years old when samples were taken; Patient 2 was 35–41 years old while enrolled in this study.

### Neutrophil Handling and Enrichment

Whole blood of patients was overlaid on a diluted Ficoll gradient (Ficoll in PBS, 5:1) as described (17). Healthy controls (HCs) (shipment and local controls) were processed with diluted (HC) and undiluted Ficoll [polymorphonuclear (PMN)-undil] and were always run in parallel. The bottom of the gradient was collected, and red blood cells (RBCs) were lysed in a buffered ammonium chloride solution (RBC lysis buffer, eBioscience). The PMN cells were then directly used for functional analysis or RNA extraction, fixed in paraformaldehyde (PFA) for electron microscopy analysis, or frozen in liquid nitrogen for proteomic analysis. Importantly, FACS staining on HC neutrophil pellets confirmed a strong enrichment of neutrophils (Figures S1A,B in Supplementary Material).

### Genetic Analysis

DNA was extracted from whole blood and subsequently *CEBPE* gene was analyzed *via* capillary sequencing as previously described (22). The following primers were used: Exon 1.1: forward 5′-CAGGCCCAGGTCAGGAG, reverse 5′-GGGCTGCTGTAGATGCCAG; exon 1.2: forward 5′-CTCTTTGCCGTGAAGCCAG, reverse 5′-CTCAGCAGCATGAGCCG; exon 2: forward 5′-GACGCATCAAGTGTGCCC, reverse 5′-TCCATGGTCTATGTCTCAGGG.

### Proteomic Analyses

Neutrophil pellets were obtained as described earlier. Two neutrophil pellets of each analyzed individual were separately subjected to lysis in Frackleton buffer (10 mM Tris–HCl, pH 7.5; 50 mM NaCl; 30 mM NaPPi; 50 mM NaF, 1% Triton X-100) supplemented with a protease inhibitor cocktail containing AEBSF, Aprotinin, Bestatin, E-64, Leupeptin, and Pepstatin A (Sigma-Aldrich, Austria) for 20 min on ice. 30 µg protein was loaded onto an SDS-PAGE gel (Novex^®^ NuPAGE^®^ 4–12% Bis–Tris Gel, Thermo Fisher) that was subsequently stained with Coomassie after protein separation. Each lane was sliced in 10 pieces (Figure S1C in Supplementary Material). Proteins in each slice were alkylated and digested *in situ* with trypsin (Promega, USA) over night. Peptides were purified with C18 extraction disks (3 M, St. Paul, MN, USA) and eluted with 5% formic acid for subsequent analysis on a nano-HPLC system (Agilent Technologies) coupled to an LTQ Orbitrap Velos (Thermo Fisher).

### Immunoblots

Immunoblots were performed as described in Ref. (23) with minimal modifications. Antibodies used were directed against CEACAM1 (clone tsg101, gift of Peter Draber, Institute of Molecular Genetics of the ASCR, Prague, Czech Republic), pan-CEACAMs (clone AG11) (24), lactoferrin [polyclonal antibody (pAb) A0186, DAKO, Denmark], MPO (pAb, A0398, DAKO, Denmark), and actin (pAb, A2066, Sigma-Aldrich, Germany), CD13 (clone BF-10, Santa Cruz, CA, USA). For gp91phox, p22phox, LCN2, and GAPDH immunoblot neutrophil pellets were obtained as described in neutrophil handling and enrichment and then lysed in Frackleton buffer for 15 min on ice supplemented with protease inhibitor cocktail (Sigma-Aldrich). Antibodies used were directed against gp91phox (ab129068, Abcam), p22phox (CS9, Santa Cruz Biotechnology), LCN2 (D4M8L, Cell Signaling Technology), and GAPDH (6C5, Santa Cruz Biotechnology). Immunodetection was performed using secondary horseradish peroxidase-coupled goat anti-mouse (554002, BD Biosciences) or goat anti-rabbit (172–1019, BioRad) secondary antibodies with ECL Prime (Amersham).

### Electron Microscopy

Samples were processed and analyzed essentially as described in Ref. (24). For immunoEM, 9–13 sections were analyzed per individual.

### Immunofluorescence Microscopy

After RBC lysis, cell pellets were resuspended in cold PBS at 1.5 million cells/mL and let attach to adhesion slides (Marienfeld, Germany) according to the manufacturer’s instructions or to poly-l-lysine-coated coverslips. Cells adhered to adhesion slides were fixed 10 min at 4°C followed by 5 min at room temperature in 3.5% PFA in PBS. Blocking was performed 30 min, in Fc Receptor Block (Innovex Biosciences, USA) and 30 min, in 10% goat serum in PBS. Antibodies were incubated in 1% goat serum in PBS. Before staining with lectins, slides were additionally blocked with Streptavidin/Biotin-blocking kit (Vector Laboratories, USA). Cells adhered to poly-l-lysine-coated coverslips were fixed for 20 min in 4% PFA/PBS solution, permeabilized with 1% Triton X-100, and blocked on 1% BSA in PBS supplemented with 0.1% Triton X-100. Antibodies directed against C/EBPε were incubated over night at 1:50 (sc-515192, mouse monoclonal, Santa Cruz) or 1:35 (HPA002928, rabbit polyclonal, Sigma-Aldrich) dilution; against p22phox (CS9, Santa Cruz Biotechnology) at 1:200 dilution. Species-specific secondary antibodies coupled to Alexa Fluor 488 were used. Cells were counterstained with DAPI. Other antibodies and lectins used for immunofluorescence and immunoelectron microscopy were as follows: antibodies directed against lactoferrin (pAb, A0186, DAKO, Denmark), MPO (pAb, A0398, DAKO, Denmark), CD15 (clone 4D1/VIMD5, gift of Johannes Stöckl, Institute of Immunology, Medical University of Vienna, Austria), CD15s (clone 2H5, BD Biosciences, USA), lysozyme (clone LZ-2, An Der Grub Bioresearch, Austria) and the lectins peanut agglutinin (PNA; from *Arachis hypogea*), and *Ulex europaeus* agglutinin I (UEA-I) (Vector Laboratories, USA) were purchased. Streptavidin or goat anti-rabbit IgG, goat anti-mouse IgG, and goat anti-mouse IgM antibodies directly linked to Alexa488 or Alexa546 (ThermoScientific, USA) were used as required. Images were analyzed with Fiji.

### Flow Cytometry

Whole blood was mixed with N-Formyl-Met-Leu-Phe (fMLF) (Glycotope, Germany) at a final concentration of 5 nM and incubated for the indicated time at 37°C. Stimulation was stopped by plunging the cells into ice-cold RBC lysis buffer in 20× excess and subsequent centrifugation. After complete RBC lysis, cells were fixed or not, in cold PFA 3.5% and permeabilized in Reagent B (Fix&Perm kit, An Der Grub Bioresearch, Austria). Samples were analyzed as described (25). Diagnostic immunophenotype was assessed as previously published (26, 27).

Polymorphonuclear cells subjected to CD14 and CD15 surface staining were derived as described in neutrophil handling and enrichment and taken up in RPMI 1640 medium (Gibco) supplemented with 10% fetal bovine serum, sterile-filtered (Sigma-Aldrich). Cells were stained with anti CD14-PC7 (RMO52, Beckman Coulter) and anti-CD15 (SSEA-1)-FITC (HI98, BioLegend) antibody at room temperature for half an hour. Cells were washed once with PBS immediately analyzed by flow cytometry.

### Analysis of Oxidative Burst

Oxidative burst was assessed as previously described (28). For analysis, unstimulated cells were used to set the gate for AlexaFluor 488 positivity.

### RNA Expression Analysis

RNA of enriched neutrophils was extracted with the RNeasy^®^ kit (Qiagen). cDNA conversion was done with the M-MLV reverse transcriptase (Promega) according to the manufacture’s protocol with 500 ng RNA as starting material. Quantitative analysis was done with the KAPA SYBR^®^ FAST ABI PRISM^®^ polymerase on a StepOnePlus^TM^ Real-Time PCR System from Applied Biosystems. Primers were designed so that they only amplify cDNA and tested on HC gDNA for unspecific products. Following primers were used: *LTF*-forward 5′-AGGAAAAGTGAGGAGGAAGTGG-3′, *LTF*-reverse 5′-TGGCATCAGCTTCTCCTTTCA-3′; *LCN2*-forward 5′-CACCCTCTACGGGAGAACCA-3′, *LCN2*-reverse 5′-GGTCGATTGGGACAGGGAAG-3′; *ALOX15*-forward 5′-ACAGACGTGGCTGTGAAAGA-3′, *ALOX15*-reverse 5′-AAAGAGACAGGAAACCCTCGG-3′; *OLFM4*-forward 5′-GGAGGTGGAGATAAGAAATATGAC-3′ *OLFM4*-reverse 5′- GACGGTTTGCTGATGTTCAC-3′; *VIM*-forward 5′-GTGGACCAGCTAACCAACGA-3′, *VIM*-reverse 5′-CCTGGATTTCCTCTTCGTGGA-3′; *SYNE2*-forward 5′-GAATGAAACCTCTGCCTGTG-3′; *SYNE2*-reverse 5′-CGATTTCTCCCTGTAGTTGTG-3′; *MYO18A*-forward 5′-CTTCACCAAGAAACGGCTCC-3′: *MYO18A*-reverse 5′-CTCACTGTCAAACCTCCTCTG-3′; *CEACAM1*-forward 5′-GATCATAGTCACTGAGCTAAGTC-3′; *CEACAM1*-reverse 5′-TTTACGTTCAGCATGATGGG-3′; *HPRT1*-forward 5′-CCCTGGCGTCGTGATTAGTG-3′; *HPRT1*-reverse 5′-TCGAGCAAGACGTTCAGTCC-3′.

### Chemotaxis Assay

Neutrophil chemotaxis was assessed using HTS Transwell-96 Permeable Support with 5.0 µm pore polycarbonate membrane (Corning). Per measurement, 50,000 neutrophils were placed in the upper chamber and incubated for 20 min at 37°C. The number of migrated neutrophils toward 5 nM fMLF (Glycotope, Germany) placed in the lower chamber was assessed using flow cytometry.

### Labeling of *S. aureus*

*Staphylococcus aureus* (strain USA300) grown in tryptic soy broth medium was provided by Philipp Starkl, PhD (laboratory of Sylvia Knapp, Research Center for Molecular Medicine of the Austrian Academy of Sciences). After washing in PBS, bacteria were heat inactivated at 65°C for 45 min. Heat inactivated bacteria were taken up in pHrodo Red, SE (Invitrogen) dissolved in 0.1 M NaHCO_3_ and then incubated for 1 h at 37°C. Labeled bacteria were resuspended in the appropriate volume of PBS for a final CFU of 1 × 10^10^/mL as determined by sequential dilutions of *S. aureus* prior heat inactivation.

### Phagocytosis Assay

Prior phagocytosis heat inactivated, pHrodo red labeled *S. aureus* was opsonized in RPMI 1640 medium (Gibco) supplemented with 15% autologous serum for 1 h at 37°C. Autologous serum was freshly isolated from whole blood using S-Monovette Z-Gel (Sarstedt) according to the manufacturer’s instructions. Opsonized bacteria were incubated with neutrophils at MOI of 50 at 37°C in the water bath for 0, 30, and 60 min. Post incubation phagocytosis was stopped by washing cells in ice-cold PBS. Immediately before measurement trypan blue was added to cell suspension and phagocytosis was assessed by flow cytometry.

## Results

### Case Reports

**Figure 1:**
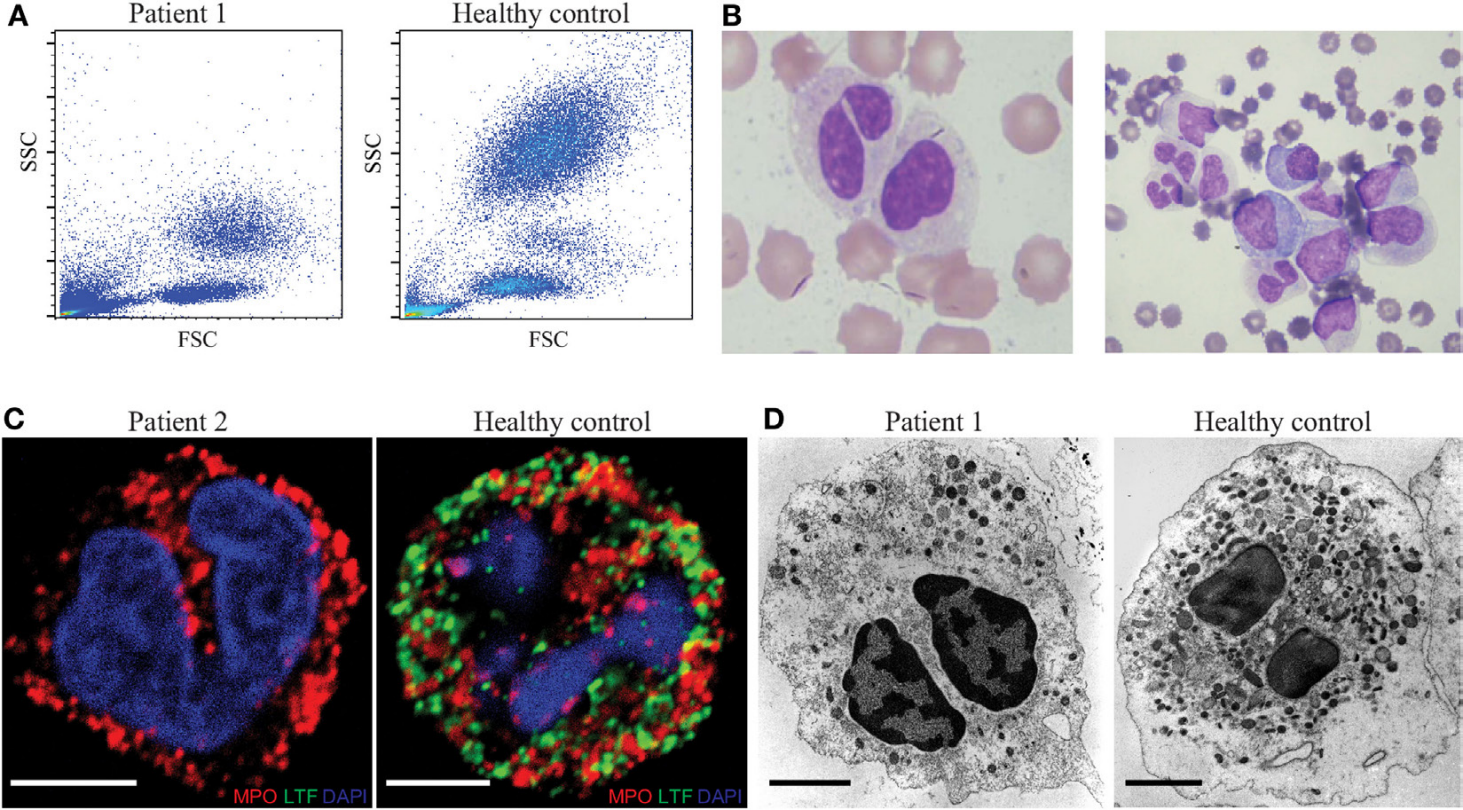
Characteristic aberrations in neutrophil granulocytes from patients with specific granule deficiency. **(A)** Forward/sideward scatter of whole blood after erythrocyte lysis. **(B)** Hematoxylin/eosin staining of peripheral blood smear (left) and bone marrow aspirate (right) of the patient. **(C)** Immunofluorescence analysis of myeloperoxidase (MPO, red) and lactoferrin (LTF, green) in the neutrophils of patient (left) and healthy donor (right), scale bar: 5 µm. **(D)** Analysis of patient (left) and healthy control (right) neutrophils in transmission electron microscopy; scale bar: 2 μm.

We studied an index patient P1 who presented first at the age of 3 months with life-threatening orbitocellulitis and sepsis. The white blood count in acute disease phase revealed leukocytosis (up to 50,000/μl) with predominant myelomonocytic cells and immature blasts, which resolved after successful treatment of the sepsis. Flow cytometric analysis of peripheral blood and bone marrow revealed a severe reduction of neutrophil granularity, which results in clustering of neutrophils with monocytes in the forward sideward scatter (Figure 1A). Morphologically, neutrophils presented hyposegmented with bilobed nuclei (Figure 1B, left panel) which was confirmed in bone marrow aspiration (Figure 1B, right panel). Family history revealed that the mother (referred to as patient P2) had frequent bacterial respiratory tract infections during childhood. Analysis of P2’s neutrophils revealed the same phenotypical abnormalities. The protein lactoferrin, which is normally present in secondary granules of neutrophils, was undetectable in patients’ neutrophils (Figure 1C). Electron microscopic analysis revealed a reduction in the number of granules in patients’ neutrophils in comparison with HCs (Figure 1D). Neutrophil chemotaxis and phagocytosis were unaltered in the patients (Figures S3C,D in Supplementary Material), further no difference in apoptosis could be detected (data not shown). Based on these findings, the two patients were assumed to have SGD.

### Identification of Mutations in *CEBPE* as Disease Etiology

**Figure 2:**
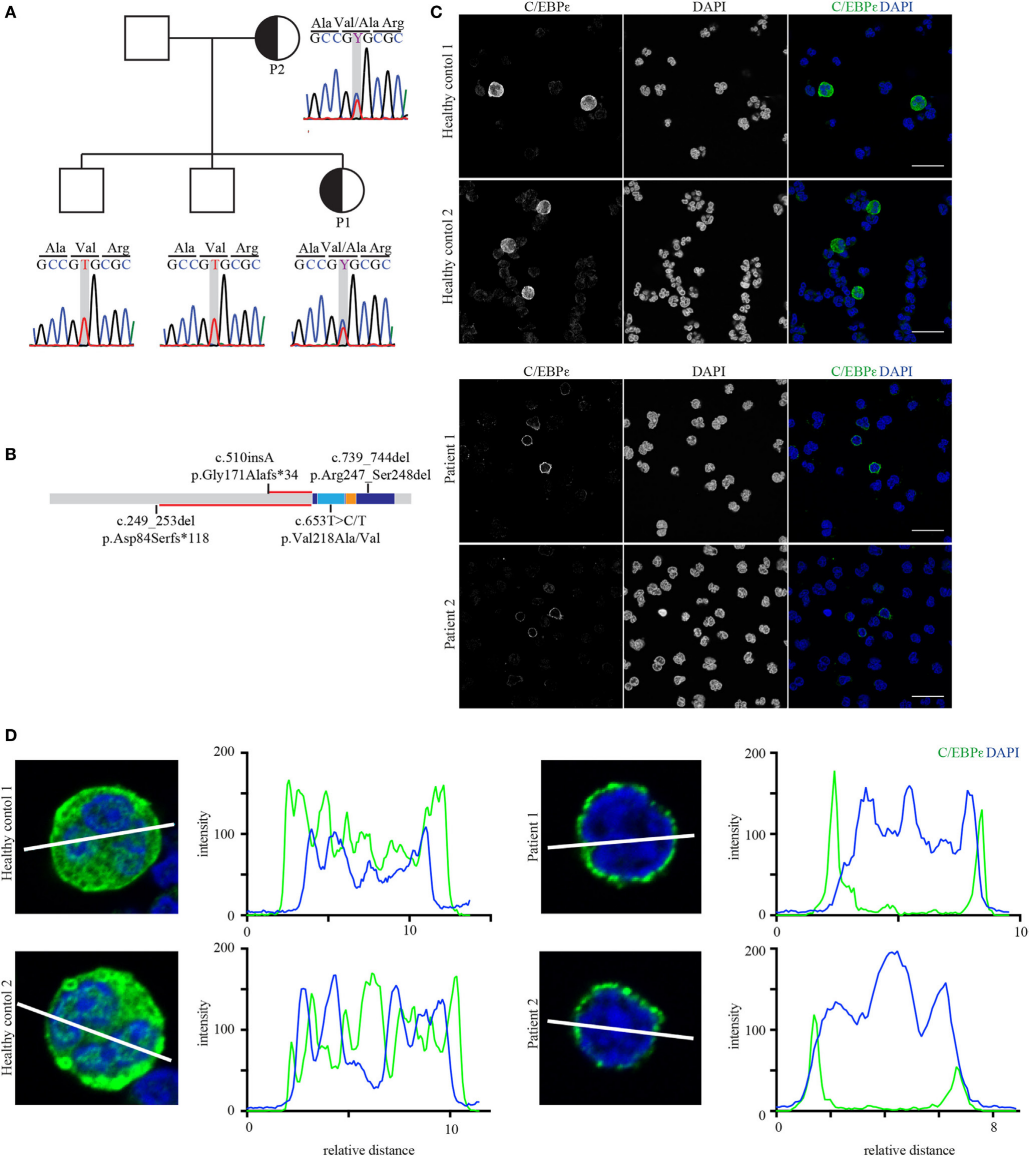
The affected mother and child both carry the heterozygous mutation p.Val218Ala in *CEBPE*. **(A)** Sanger chromatograms of the core pedigree covering the mutated nucleotide (framed gray) in *CEBPE*. **(B)** Protein model including domain structure of C/EBPε (basic leucine zipper: dark blue; basic subunit: light blue; and leucine zipper: orange) showing the relative location of the identified mutation in relation to the homozygous frameshift mutations. The length of the altered reading frames is indicated in red. **(C)** Confocal images of healthy control and patient granulocytes stained for C/EBPε (green, scale bar: 20 µm). Stains were performed in triplicates. **(D)** Line graphs of C/EBPε and DAPI show that in patients’ cells the center of the nucleus lacks C/EBPε which rather localizes to the perinuclear region (blots were done with ImageJ).

At the time of investigation, mutations in the gene *CEBPE* were the only known cause for SGD, defining the molecular etiology in the majority of patients (9, 15–17). Sanger sequencing of the two exons of this gene revealed a heterozygous missense mutation resulting in an amino acid exchange at position 218 from valine to alanine (c.653C/T>C; p.Val218Ala; Figure 2A). This mutation has been already described in another single patient (17). The mutation showed perfect segregation with the disease, with both affected individuals carrying the mutation in a heterozygous state while the non-affected brothers of P1 were wild type (Figure 2A). The mutation lies within the basic subunit of the b-zip domain, which is responsible for DNA–protein interaction (Figure 2B). As the identified mutation caused complete absence of the protein product of the known C/EBPε-target gene *LTF* (Figure 1C), we were interested how the Val218Ala-mutated C/EBPε prevents the binding of the remaining wild-type C/EBPε to the corresponding promotors. To elucidate this, we performed confocal microscopy of HC and patient-derived granulocytes and observed that mutant C/EBPε-expressing cells present a perinuclear location of C/EBPε as shown by confocal imaging (Figures 2C,D) providing a potential explanation for the complete absence of lactoferrin in our initial analysis.

### Neutrophil Granules in SGD Are Misdistributed, Undergo a Change in Protein Composition, and Lack Several Glycoepitopes

**Figure 3:**
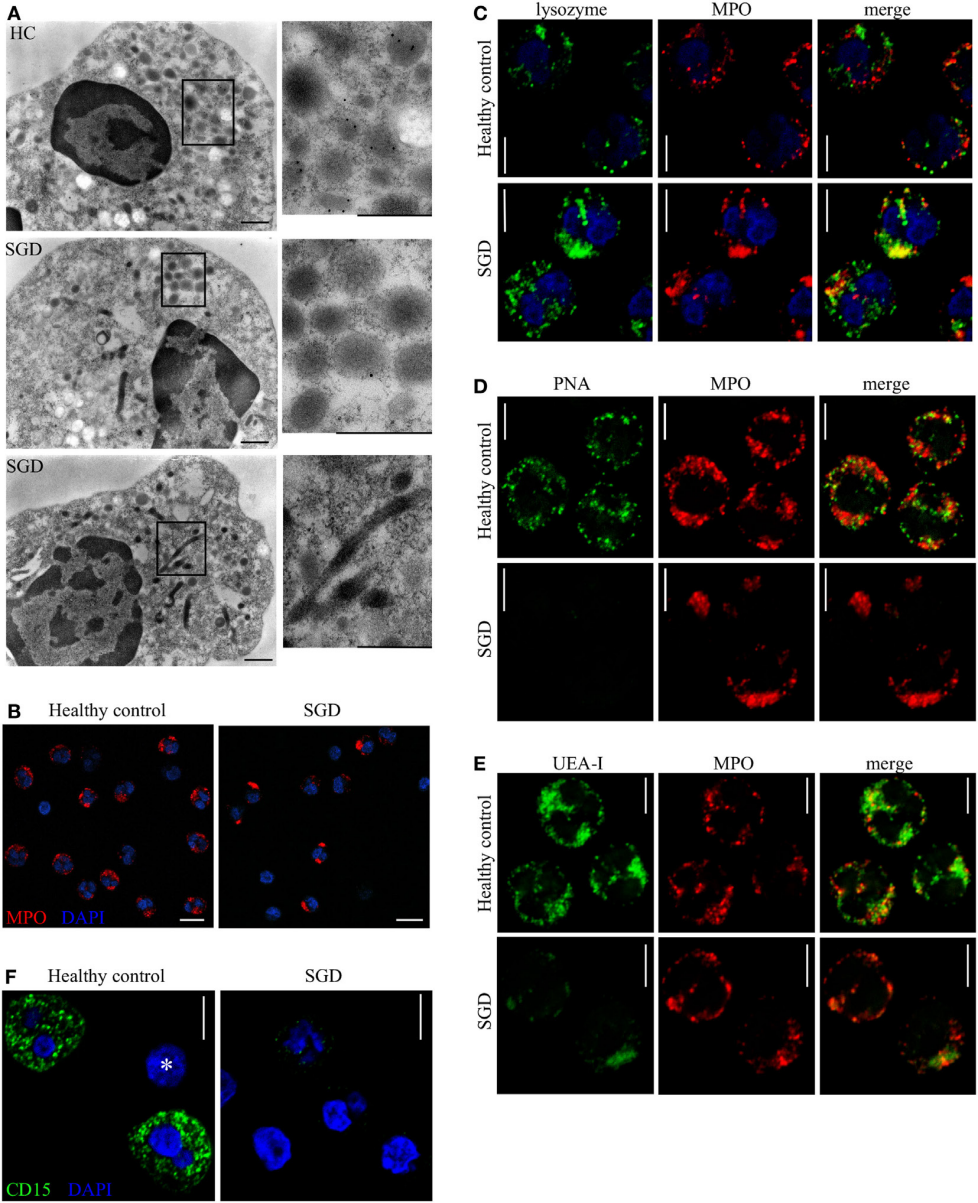
Granule distribution, protein content, and glycosylation pattern of neutrophil granules are modified in specific granule deficiency (SGD). **(A)** Immunoelectron microscopy shows presence of lactoferrin (15 nm gold particles) in secondary granules in healthy controls (HCs, upper panel) and its absence in two SGD patients (two lower panels). Elongated sacs as well as aggregates of granules are observed in SGD but not in HCs (see zoomed in panels). Scale bars: 500 nm. **(B)** MPO-containing granules (red) are aggregated and polarized in SGD compared with HCs as shown by confocal immunofluorescence. Scale bar: 10 µm. **(C)** Lysozyme (green) co-localizes with MPO (red) in neutrophils of SGD patients (lower panel) but not of HCs (upper panel). **(D,E)** Different populations of granules are recognized specifically by the lectins (green) peanut agglutinin (PNA) **(D)** and UEA-I **(E)** in HCs (upper panel), displaying, respectively, no or partial co-localization with MPO (red). The granules in SGD neutrophils (lower panel) are not recognized by PNA **(D)** and react weakly with *Ulex europaeus* agglutinin I (UEA-I) **(E)** compared with HCs. **(F)** The anti-CD15 (green) staining is reduced in neutrophils of SGD patients (right) compared with healthy donor (left). No staining is visible in a lymphocyte in the healthy donor (asterisk). Scale bars **(C–F)**: 5 µm.

In line with previous studies (7, 8, 29), findings from transmission electron microscopy illustrated a reduced number of neutrophil granules in both patients with SGD (Figure 1D). Two studies indicate that the disease results in the reorganization of granules and not solely in the absence of granules. The different granule populations undergo a change in buoyancy upon separation on a Percoll gradient (30) and elongated peroxidase-negative granules have been observed by electron microscopy (29). We characterized further the intracellular structures assembled during the abnormal neutrophil differentiation in SGD. We applied immunoelectron microscopy on patients’ granulocytes. As predicted, we hardly detected any lactoferrin in patients’ neutrophils in contrast to HCs (Figure 3A, upper and middle panel; Figure S2A in Supplementary Material). However, small, ovoid granules resembling secondary granules were still observed in addition to bigger round granules, classically identified as primary granules (Figure 3A, middle panel). Thus, from our data, it is not possible to conclude on the complete absence of all specific-like granules in neutrophils in SGD.

In our study, neutrophils from the patients’ revealed two additional, abnormalities not described previously. We noticed elongated sacs with internal membranous structures reminiscent of the cisternae of mitochondria (Figure 3A, lower panel). These structures were larger than the abnormal granule structures observed in a previous case of SGD (11). Furthermore, we observed that in more than half of the cells, the remaining granules are not evenly distributed and group together in clusters. These clusters contain various types of vesicles, most of which are big and round, corresponding to primary granules. We subsequently analyzed the vesicles contained in those clusters with immunofluorescence. MPO was concentrated at those sites (Figure 3B). Furthermore, MPO and lysozyme co-localized extensively in neutrophils from patients whereas in HCs both proteins were localized to distinct granules (Figure 3C). In absence of intact secondary granules, lysozyme is targeted mainly to the primary granules, resulting in change in the protein composition of these granules.

Although the secondary and tertiary granules on neutrophils are known to bear the carbohydrate epitope CD15 (Lewis X), the distribution of glycoepitopes in neutrophil granules has not been fully characterized to date. We found that two lectins, PNA (from *A. hypogea*) and UEA-I, labeled granular structures in neutrophils of HCs but faintly stained patients cells (Figures 3D,E). In HCs, the staining with PNA (Figure 3D) did not co-localize with either MPO (Figure 3D) or lactoferrin (Figure S2C in Supplementary Material) and the staining with UEA-I (Figure 3E) partially co-localized with MPO (Figure 3E) and lactoferrin (Figure S2B in Supplementary Material). The intracellular staining intensity for CD15 (Lewis X) was reduced in patients neutrophils (Figure 3F). No difference was observed for the CD15s (sialyl Lewis X) epitope (Figure S2D in Supplementary Material). In sum, the lectins PNA and UEA-I specifically recognize granules in neutrophils as demonstrated by the absence of binding in patients’ cells. Interestingly, those two lectins stain different compartments than secondary granules in HCs, suggesting that other cellular compartments may also be altered in neutrophil in SGD. Indeed, UEA-I positive granules are partially MPO-positive and lactoferrin-negative, PNA-positive granules are MPO-negative, lactoferrin-negative while CD15 is known to co-localize with lactoferrin. Therefore, novel glycoepitopes characterize different granule populations which are absent in SGD.

### Altered Surface Marker Expression in SGD

**Figure 4:**
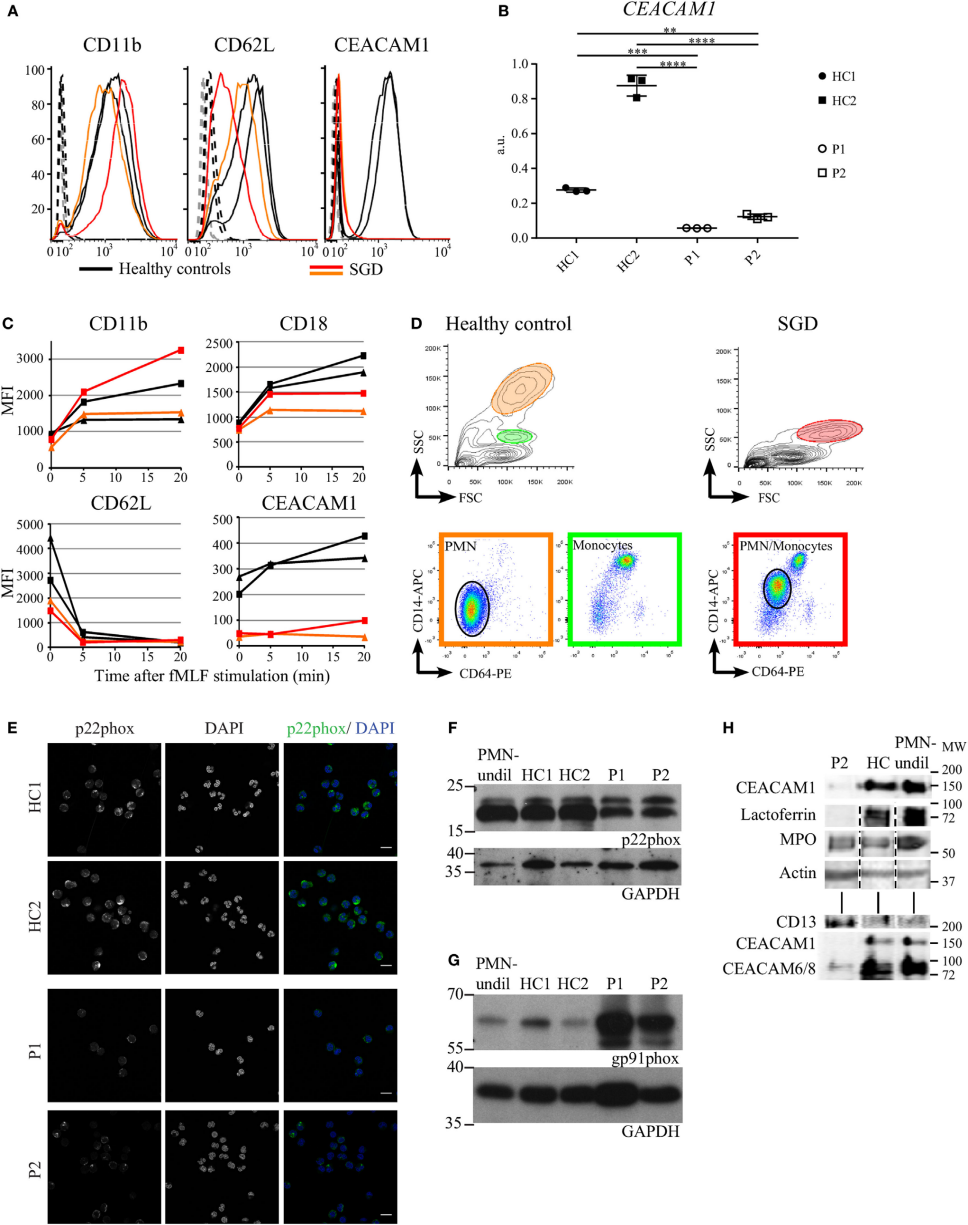
Targeted analysis of the expression of surface and granule proteins in neutrophils of specific granule deficiency (SGD) patients **(A)**. Total protein levels of CD62L, CD11b, and CEACAM1 measured by flow cytometry in fixed and permeabilized myeloid cells in P1 (red) and P2 (orange) affected by SGD compared with two healthy controls (HCs) (black). The isotype controls for the SGD and normal cells are displayed as dashed black and gray curves, respectively. **(B)** *CEACAM1* transcript levels in neutrophils were assessed in both patients (P1 and P2) and two HCs (HC1, shipment control; HC2, local control) and revealed a significant reduction of transcript levels as tested with one-way ANOVA (Bonferroni corrected for multiple testing, normalization to *HPRT1* expression, three biological replicates shown). **(C)** Changes in mean fluorescence intensity (MFI) induced by fMLF stimulation (5 nM for 5 or 20 min) on the surface of live myeloid cells in P1 (red) and P2 (orange) affected by SGD compared with two HCs (black). As the monocytes and polymorphonuclear cell populations cannot be separated on the FSC/SSC channels in the SGD sample, the MFI is calculated as an average of both cell populations. **(D)** Elevated surface expression of CD14 and CD64 in live polymorphonuclear cells (PMNs) in SGD (right) compared with HCs (left). In the upper FSC/SSC panels, PMN (orange circle) and monocyte (green circle) populations are visible in the HCs, but these populations cannot be unmixed in the SGD sample (red circle). In the lower panel, the CD64-PE/CD14-APC stain distinguishes the monocytic CD64^high^/CD14^high^ population (green panel) from the PMN CD64^low^/CD14^low^ (orange panel, black circle). These populations are found back in SGD (red panel), and a PMN population is identifiable (black circle). The expression of both CD64 and CD14 is clearly higher in the PMN population in SGD versus healthy donors. **(E)** Immunofluorescence analysis of p22phox (green) in neutrophils of patients (P1, P2) and healthy controls (HCs) (HC1, local control; HC2, shipment control). Scale bar: 10 µm. **(F,G)** Analysis of p22phox **(F)** and gp91phox **(G)** by Western blot. **(H)** The amount of specific granule markers in a total cell lysate was compared by immunoblotting. The signal for actin serves as a loading control. The SGD and HC fractions were enriched for PMNs on a modified Ficoll gradient, and erythrocytes were removed by lysis as described. The PMN fraction contains >98% PMNs, separated on a Polymorphprep gradient.

Regulated exocytosis of preformed receptors enclosed in granules to the cell surface is essential for normal cell function. The complement receptor 3, composed of the CD11b and CD18 integrins, and CEACAM1 are part of the membrane coat of secondary granules and are released to cell surface upon fMLF stimulation in healthy individuals (1). Thus, we monitored the expression levels of these proteins and their mobilization to the cell surface in myeloid cells of SGD patients and used the shedding of CD62L from the cell surface as a control of cell activation kinetics. Despite the absence of secondary granules, the total expression level of CD11b revealed no difference in SGD patients (Figure 4A). However, CD62L expression was reduced. Furthermore, CEACAM1 was not detectable in the patients as assessed by FACS and qPCR (Figures 4A,B). Of note, *CEACAM1* transcript levels of patient neutrophils were compared with two HCs with HC1 being a shipment control and HC2 a local control. Importantly, handling of samples during the shipment already dramatically reduced *CEACAM1* transcript levels, however, both patients present with significantly less *CEACAM1* levels in comparison with both HCs (Figure 4B). The surface expression of CD11b, CD62L, and CEACAM1 in living cells (Figure 4C) correlates with total expression levels (Figure 4A). Upon stimulation with fMLF, the mobilization of CD11b to the cell surface and the shedding of CD62L are comparable in HCs and patients. We detected a slight reduction of CD18 cell surface recruitment whereas CEACAM1 surface expression remained absent in patients (Figure 4C).

Flow cytometry analyses of blood cells of SGD patients and HCs revealed further differences in surface marker expression (Figure 4D). When stained for the monocyte markers CD14 and CD64, monocytes can be clearly separated from granulocytes although a majority of the granulocytes in patients display higher CD14 and CD64 expression levels.

Specific granule deficiency patients present with reduced activity of the neutrophil NADPH oxidase following stimulation with zymosan (31–33). To investigate the expression of p22phox, one of the membrane components of the NADPH oxidase, we performed an immune fluorescence analysis, which revealed that patient-derived cells present reduced but not absent expression (Figure 4E). These data were confirmed with Western Blot (Figure 4F). Interestingly, gp91phox expression, was elevated in SGD neutrophils when compared with HCs (Figure 4G). Thus, NADPH oxidase is still present in SGD patients. Accordingly, oxidative burst into the phagocytic vacuole was intact in neutrophils derived from SGD patients (Figures S3A,B in Supplementary Material). Of note, chemotaxis and phagocytosis of CEBPE-mutant neutrophils were also not affected (Figures S3C,D in Supplementary Material).

### Reduced Expression of Granule Proteins and Increased Expression of Proteins Controlling Nuclear Shape in SGD Neutrophils

We wanted to understand whether the *CEBPE* mutation only affects the expression of proteins targeted to the secondary granules. Thus, we monitored the protein expression levels of classical markers of distinct granule populations by immunoblotting of total cell lysates (Figure 4H). We found that levels of MPO, a marker of primary granules, were comparable in HCs and patients, whereas lactoferrin, as seen also with other methods, was absent. Interestingly, CEACAMs expression was reduced confirming the data seen in FACS and qPCR analysis (Figures 4B,C). The expression level of CD13, as a marker of secretory vesicles, was increased in patients’ cells. Actin served as a loading control (Figure 4H). These data were confirmed with flow cytometry (data not shown).

**Figure 5:**
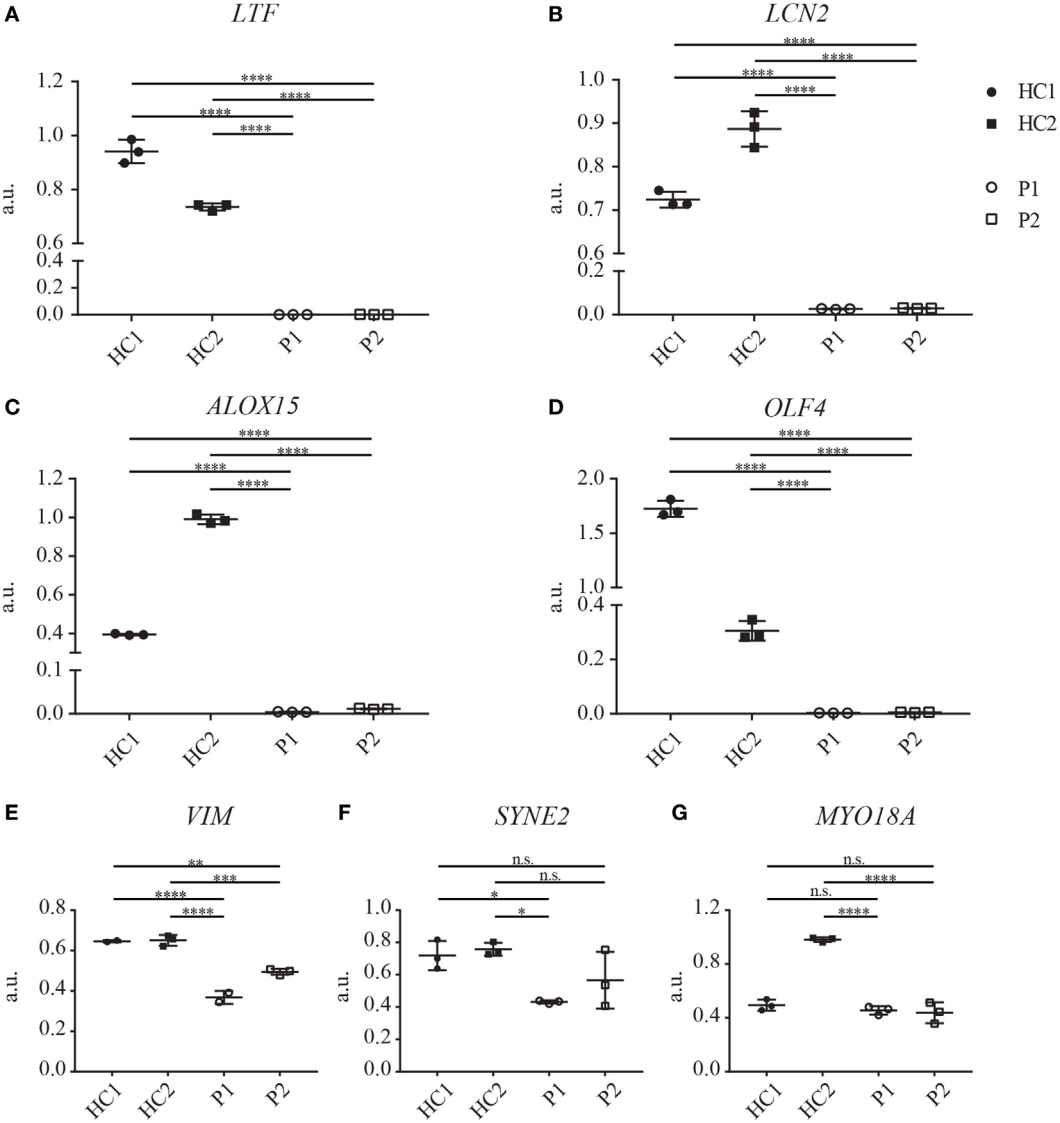
mRNA expression of several granule proteins in neutrophils of specific granule deficiency patients is reduced. **(A–G)** Transcript analysis of *LTF* **(A)**, *LCN2* **(B)**, *ALOX15* **(C)**, *OLF4* **(D)**, *VIM* **(E)**, *SYNE2* **(F)**, and *MYO18A* **(G)** on neutrophils of both patients (P1 and P2) and two healthy controls (HC1, shipment control; HC2, local control, three biological replicates shown). Gene expression was normalized to *HPRT1* expression. Significance levels were determined with one-way ANOVA (Bonferroni corrected for multiple testing).

To extend our analysis of the impact of the here described *CEBPE* mutation on protein expression in neutrophils, we performed a proteomics analysis on total cell lysates from enriched fraction of PMNs from patients and HCs (isolated with modified and normal Ficoll density). Overall, we identified more than 2,000 proteins in the neutrophil-enriched fraction (Table S1 in Supplementary Material). We compared absence or presence of proteins in control and SGD neutrophils semiquantitatively, by analysis of the respective spectral counts. Decreased expression of more than 10-fold or increased expression of more than 20-fold in SGD sample was regarded as relevant. This analysis revealed 38 proteins with altered protein expression in SGD neutrophils compared with healthy volunteers (Tables 1 and 2). Among the proteins that were absent or reduced in patient neutrophils, the majority (18/20) have been shown to localize to secondary/tertiary granules in PMNs. This includes proteins abundant in the matrix of secondary granules [lactoferrin (LTF), neutrophil gelatinase-associated lipocalin (LCN2), transcobalamin-1, and olfactomedin-4 (OLF4)], tertiary granules (neutrophil collagenase, arginase-1) and subtypes of primary granules (neutrophil defensin 1) in neutrophils and in the matrix of eosinophilic granules (arachidonate 15-lipoxygenase (ALOX15), eosinophil major basic protein homolog/proteoglycan 3) in SGD (Table 1). We identified two additional proteins that were not associated with granule function—namely, the oxidized low-density lipoprotein receptor 1 and copine-2. Both have been shown to be expressed in neutrophils (34, 35). Analysis of the mRNA expression of the top four candidates *LTF, LCN2, OLF4*, and *ALOX15*, and protein expression of LTF and LCN2 revealed a severe reduction or even absence of the transcripts (Figures 4H and 5A–D; Figure S3E in Supplementary Material) suggesting that they are direct targets of C/EBPε.

**Table 1:** Total cell lysates of myeloid cells from specific granule deficiency (SGD) patients and healthy controls (HCs) and a polymorphonuclear (PMN)-undil fraction of HCs were prepared as in Figures 4F,G.

Protein name	Uniprot entry name	Spectral counts SGD	Spectral counts HC	Spectral counts PMN-undil	Localization in granules
Lactotransferrin	TRFL_HUMAN	0 (0/0)	1,228 (1,110/1,346)	1,130 (1,173/1,087)	Secondary
Neutrophil gelatinase-associated lipocalin	NGAL_HUMAN	0 (0/0)	130 (137/123)	225.5 (226/225)	Secondary
Arachidonate 15-lipoxygenase	ALOX15_HUMAN	0 (0/0)	76.5 (89/64)	45.5 (40/51)	Eosinophils
Olfactomedin-4	OLFM4_HUMAN	0 (0/0)	31 (31/31)	96 (102/90)	Secondary
Cathelicidin antimicrobial peptide	CAMP_HUMAN	0 (0/0)	28.5 (22/35)	47.5 (43/52)	Secondary
Arginase-1	ARGI1_HUMAN	0 (0/0)	25 (29/21)	18 (18/18)	Tertiary
Chitotriosidase-1	CHIT1_HUMAN	0 (0/0)	20 (18/22)	73 (75/71)	Secondary
Chitinase-3-like protein 1 (YKL-40)	CH3L1_HUMAN	0 (0/0)	18.5 (22/15)	20.5 (17/24)	Secondary
Proteoglycan 3	PRG3_HUMAN	0 (0/0)	14 (16/12)	10 (8/12)	Eosinophils
Transcobalamin-1	TCO1_HUMAN	0 (0/0)	13.5 (16/11)	23.5 (25/22)	Secondary
Cysteine-rich secretory protein 3	CRIS3_HUMAN	1 (2/0)	20 (22/18)	18.5 (18/19)	Secondary
Bactericidal permeability-increasing protein	BPI_HUMAN	11 (10/12)	184.5 (182/187)	198 (209/187)	Primary
Eosinophil lysophospholipase	LPPL_HUMAN	4 (3/5)	56.5 (54/59)	43 (36/50)	Eosinophils
Oxidized low-density lipoprotein receptor 1	OLR1_HUMAN	0.5 (1/0)	6.5 (9/4)	13.5 (16/11)	Unknown
Neutrophil collagenase	MMP8_HUMAN	10 (8/12)	124 (105/143)	121.5 (129/114)	Secondary
Neutrophil defensin 1	DEF1_HUMAN	3 (2/4)	37 (33/41)	57.5 (61/54)	Primary
Peptidoglycan recognition protein 1	PGRP1_HUMAN	2 (1/3)	24 (24/24)	39 (37/41)	Tertiary
Copine-2	CPNE2_HUMAN	1 (0/2)	10.5 (12/9)	15 (16/14)	Unknown
Haptoglobin-related protein	HPTR_HUMAN	3.5 (3/4)	35 (36/34)	36.5 (35/38)	Tertiary

*Samples were loaded onto a polyacrylamide gel that was subsequently sliced into 10 pieces that were individually analyzed by liquid chromatography mass spectrometry*.

*Experiments were performed in biological duplicates. The average spectral count is semiquantitatively correlated to protein abundance. Spectral counts for each duplicate are indicated in brackets*.

Interestingly, we could also identify several proteins that were present in the diseased neutrophils but absent in healthy donors (Table 2). A classification of these proteins according to protein function revealed an enrichment of proteins with a role in RNA processing and translation, cell differentiation and regulation of nuclear shape. Strikingly, the expression of several members (nesprin-2, vimentin, and lamin-B2) of the linker of nucleoskeleton and cytoskeleton (LINC) complex was higher in the patient cells (Table 2). However, analysis of mRNA transcripts of the top three candidates *vimentin (VIM), nesprin-2 (SYNE2)*, and unconventional *myosin-XVIIIa (MYO18A)* did not reveal an increase of transcript presence (Figures 5E–G). Therefore, these data suggest that the expression of these proteins is posttranscriptionally regulated and that C/EBPε might elicit an indirect repressor activity on the expression of the LINC complex, a major regulator of nuclear shape (36, 37).

**Table 2:** Total cell lysates of myeloid cells from the specific granule deficiency (SGD) patients and normal donors (ND) and a polymorphonuclear (PMN) fraction of healthy controls (HCs) were prepared as for Table 1.

Protein name	Uniprot entry name	Spectral counts SGD	Spectral counts HC	Spectral counts PMN-undil	Biological process
Vimentin	VIME_HUMAN	108.5 (97/120)	0 (0/0)	0 (0/0)	Nuclear organization
Nesprin-2	SYNE2_HUMAN	55.5 (28/83)	0 (0/0)	1.5 (3/0)	Nuclear organization
Unconventional myosin-XVIIIa	MY18A_HUMAN	28 (16/40)	0 (0/0)	0 (0/0)	Intracellular trafficking
Far upstream element-binding protein 2	FUBP2_HUMAN	25.5 (17/34)	0 (0/0)	0 (0/0)	RNA processing
Monocyte differentiation antigen CD14	CD14_HUMAN	19.5 (21/18)	0 (0/0)	0 (0/0)	Cell differentiation
Type-1 angiotensin II receptor-associated protein	ATRAP_HUMAN	18 (14/22)	0 (0/0)	0 (0/0)	Intracellular trafficking
Serrate RNA effector molecule homolog	SRRT_HUMAN	14 (4/24)	0 (0/0)	0 (0/0)	RNA processing
Ribosomal protein S6 kinase alpha-3	KS6A3_HUMAN	12 (10/14)	0 (0/0)	0 (0/0)	Translation
Dedicator of cytokinesis protein 8	DOCK8_HUMAN	11.5 (7/16)	0 (0/0)	0 (0/0)	Cell differentiation
U5 small nuclear ribonucleoprotein	U520_HUMAN	11.5 (8/15)	0 (0/0)	0 (0/0)	RNA processing
Eukaryotic initiation factor 4A-III	IF4A3_HUMAN	11 (12/10)	0 (0/0)	0 (0/0)	Translation
40S ribosomal protein S4, X isoform	RS4X_HUMAN	11 (10/12)	0 (0/0)	0 (0/0)	Translation
Coiled-coil domain-containing protein 88B	CC88B_HUMAN	10.5 (11/10)	0 (0/0)	0 (0/0)	Cell differentiation
40S ribosomal protein S3	RS3_HUMAN	10.5 (10/11)	0 (0/0)	0 (0/0)	Translation
Splicing factor, proline- and glutamine-rich	SFPQ_HUMAN	10 (5/15)	0 (0/0)	0 (0/0)	RNA processing
Splicing factor 3B subunit 3	SF3B3_HUMAN	40.5 (32/29)	1 (2/0)	0 (0/0)	RNA processing
Neuroblast differentiation-associated AHNAK	AHNK_HUMAN	111 (90/132)	3 (6/0)	0 (0/0)	Unknown
Lamin-B2	LMNB2_HUMAN	20.5 (17/24)	1 (2/0)	0 (0/0)	Nuclear organization
FK506-binding protein 15	FKB15_HUMAN	10 (6/14)	0.5 (1/0)	0.5 (1/0)	Unknown

*The identified proteins are listed according to decreasing spectral count ratio (ND/SGD). Spectral counts for each duplicate are indicated in brackets*.

## Discussion

We here identified two novel cases of SGD caused by an autosomal dominant mutation in *CEBPE*. We, for the first time, provide a potential explanation why the heterozygous missense mutation in *CEBPE* causes a similar phenotype as the more common homozygous frameshift mutations. Furthermore, we here performed a detailed proteomic investigation of neutrophils derived from patients with SGD. Our comparative analysis of the proteome of PMN fractions from healthy donors and SGD individuals reveals new perspectives in neutrophil development, maturation and biology and elucidates the role of the causative gene *CEBPE* in health and disease.

The identified mutation (p.Val218Ala) is located in the b-zip motif and has already been described in another unrelated case (17), confirming the causative role of this mutation in disease onset. There is no obvious genetic link between the published case and ours. Khanna-Gupta et al. have excluded the possibility of a frequent mutation at this site. Since then, no further evidence has been published that this variant might be a common variant as it is not considered as a single nuclear polymorphism and has not been mentioned in the 1000 Genomes Project (38). Furthermore, the Exome Aggregation Consortium (ExAC) database does not list the p.Val218Ala mutation (39). According to the ExAC database, a p.Val218Gly variant has been observed once in heterozygous state in the European population (including 66,606 sequenced alleles), representing a minor allele frequency of 1.501 × 10^−5^ (39). None of the other assessed populations revealed presence of this variant (121,230 total allele counts). Whether the carrier of this variant also exhibits a SGD-like phenotype and whether C/EBPε p.Val218Gly leads to a similar disease pathology through exclusion of wild-type and mutated C/EBPε from the nucleus is beyond the focus of this study.

Analysis of the frameshift mutations uncovers a putative protein with the *CEBPE* c:249_543del mutation having 117 altered amino acids and the c.510insA mutation a 33 amino acid alteration. As both mutated proteins are in the same reading frame, they share this last part and present the same alterations. However, the altered proteins end in a newly created stop codon shortly before the b-zip domain. The recently identified homozygous in-frame deletion also affects the b-zip domain similar to the here described heterozygous mutation. Until now, it has not been clear why both dominant and recessive mutations in *CEBPE* lead to comparable phenotypes classified as SGD. Our immune fluorescence studies suggest that mutated C/EBPε prevents wild-type C/EBPε from proper localization within the nucleus, which then results in an absence of C/EBPε-activated transcripts. The regulation of C/EBPε nuclear organization and activation is barely understood. C/EBPε can be sumoylated which enhances its activity (40, 41). Protein sumoylation is associated with cellular and nuclear localization, although it is unpredictable how protein behavior is changed after sumoylation (42). On the other side, C/EBPδ, another family member of the C/EBP family of transcription factors, can interact with protein inhibitors of activated STATs γ (PIASγ) which sequesters it to the nuclear periphery, a region that is associated with reduced transcriptional activity (43, 44). It remains to be determined how the abnormal distribution of C/EBPε p.Val218Ala is triggered.

Interestingly, others (17) observe an increase of C/EBPε protein expression as detected by Western Blot. We assessed protein localization by immune fluorescence with two different antibodies raised against the first 125 amino acids. We observe an exclusion of C/EBPε from the nucleus in patient-derived cells. The increased amount of protein in Western Blot analyses suggests that a certain negative feedback loop may exist, which downregulates C/EBPε expression upon successful terminal differentiation. In line with that, previous work has shown that C/EBPε expression peaks at the myelocyte and metamyelocyte stage but is almost absent in mature cells (45). Absence of C/EBPε transcriptional activity might thus fail to downregulate its own transcription and the expression persists.

Our description of the case conveys the full picture of SGD fitting with most of the cellular defects described to date (7–9). The defects described herein were present in both the mother and child. Fitting with the clinical picture, some defects were more pronounced in the child. The patients’ neutrophils contain a reduced number of granules (Figures 1A,D). They have greatly reduced expression or lack many proteins targeted to the secondary and tertiary granules transcribed at the myelocyte stage of differentiation (Figures 1C, 3A and 4A,B; Table 1). The neutrophils also showed a reduced expression of proteins (such as BPI and HNP1) targeted to the primary granules and transcribed at the late promyelocyte stage (Table 1). Most of neutrophils display an atypical bilobed nucleus. Some proteins expressed in the matrix of secondary granules in eosinophils are lacking (Table 1), suggesting a defect in the maturation of all PMN cells (14). As almost all proteins underrepresented in patient PMNs are localized to granules, we speculate that LOX1 (known to contribute to neutrophil activation) and copine-2 (a calcium dependent membrane binding protein) are also localized to granules in neutrophils. Additionally, the absence of several other proteins in patient neutrophils opens possibilities for the development of novel marker proteins to aid in diagnosis of SGD.

Compared with normal neutrophils, the patients’ cells express higher total and surface levels of the monocytic marker CD14 (Figure 4D; Table 2). This has been described in C/EBPε knockout mice (46) and in two other cases of SGD (9, 47) and interpreted as an absence of full granulocytic differentiation. However, the neutrophils in the patients of this study also express CD64 and display reduced total and surface levels of CD62L (Figures 4A,D). CD14 and CD64 are induced by interferon γ on neutrophils (48). Increased CD14 and CD64 and decreased CD62L surface levels on neutrophils can be indicative of bacterial infections (49, 50). However, the observed differences were independent of the infection status of the patients. Some of the defects seen in other SGD cases are not detected in the here described patients, as for example, the oxidative burst and the expression levels of CD11b and CD16 are unaltered.

A reorganization of neutrophil granules rather than a mere absence of secondary granules is observed in the patients’ neutrophils. First, the intracellular organization of granules is affected as most remaining granules are clustered at a peripheral location within the cell (Figure 3A). Second, lysozyme is normally expressed throughout neutrophil maturation with a peak at myelocytic stage of differentiation (2) and accumulates to secondary/tertiary granules in neutrophils of HCs. In SGD, lysozyme is still expressed in patients’ neutrophils and accumulates in azurophil granules containing MPO (Figure 3C). A population of intermediate granules of mixed content, containing MPO and lysozyme, is observed in patients but not in HCs. Indeed, the separation of granules on a density gradient also demonstrated an abnormal distribution of markers of primary and secondary granules in SGD (30), speaking for a reorganization of granule content. Nevertheless, granule reorganization does not affect the kinetics of the early steps of neutrophil activation like the shedding of CD62L and the mobilization of CD11b^+^ granules (Figure 4C) which appear normal in patients. The comparison of the lectin profile of neutrophil identified new glycoepitopes on granules which stain differentially. Our data suggest that different glycoepitopes characterize different granule populations. Sugars recognized by PNA and UEA-I as well as the CD15 epitope itself are reduced in the patients. The altered staining in patients’ cells is subject to two explanations: either granules other than the classic lactoferrin-containing secondary granules are also missing in the patients, or several glycosylating enzymes are expressed at different levels in neutrophils of SGD and during neutrophil maturation in the bone marrow. The binding specificity of lectins on simple synthetic carbohydrates arrays is well characterized (51). PNA binds galactosylated antigens (Galβ1–3GalNAcβ) as found on the Thomsen–Friedenreich antigen (52). UEA-I binds fucosylated structures (Fucα1-2Galβ) as found on Lewis-related antigens (53). In patients affected by biallelic *JAGN1* mutation leading to severe congenital neutropenia (SCN), *N*-glycosylation of proteins in neutrophils is perturbed leading to a reduced number of galactosylated antennae and a reduced amount of antennary fucose residues (54). Intermittent signs of maturational arrest at the promyelocyte/myelocyte stage are a hallmark feature of classical SCN, and the reduced number of specific granules is observed in JAGN1-deficient patients. We hypothesize that the fucosylated and galactosylated structures, displayed on *N*-glycosylated proteins and absent in SCN, correspond to the fucose and galactose containing glycans, recognized by UEA-1 and PNA in specific granules, and absent in SGD. Strikingly, our data suggest an indirect repressor activity for C/EBPε on the expression of the LINC complex. This is an interesting consideration, as the LINC complex controls changes in nuclear shape in response to alterations in the cytoskeleton, and consistent with the observation of impaired nuclear segmentation in patient neutrophils (Figure 1B). In accordance with our data, the expression of several members of the LINC complex is suppressed in circulating granulocytes and ATRA-differentiated HL-60 granulocytes compared with immature undifferentiated HL-60 (36). It is has been shown previously that C/EBPε induces the expression of lamin B receptor (LBR). When knocked out, this leads to an aberrant nuclear phenotype similar to that observed in SGD (21). Therefore, C/EBPε might affect changes in nuclear shape by transcriptional activation of certain regulators that downregulate the protein expression of the LINC complex and upregulate LBR. In our analysis, however, we did not observe any obvious changes in LBR expression which may be consequence of our strict data filtering.

Comparison of our data (Table S1 in Supplementary Material) to the previously published transcript dataset of one SGD patient (17) revealed that most proteins, such as DEFA1, MMP8, MMP9, TCN1, and others, of which mRNA transcripts have been previously shown to be reduced, were also absent in patient neutrophils on protein level [Table S1 in Supplementary Material and Ref. (17)]. However, there were also few proteins, such as BPI and PGLYRP, which were absent in our patients, but have been shown to be upregulated on mRNA in the previously published data set [Table S1 in Supplementary Material and Ref. (17)]. So far, we do not have a definite explanation for this discrepancy, but it is important to point out that both methods have certain limitations in regards of protein/mRNA stability and detection abilities. With our comprehensive analysis of the proteome of SGD neutrophils we provide an important dataset to further study the pathological mechanisms of this disease and the biology of the transcription factor C/EBPε.

In summary, we describe here two related SGD patients whose disease is caused by heterozygous mutations (p.Val218Ala) in *CEBPE* which has been described previously (17). We further reveal that mutated C/EBPε prevents wild-type C/EBPε from translocating to the nucleus. Our detailed analysis of the protein content and cellular defects in SGD neutrophils identified a previously undescribed reorganization of neutrophil granules rather than a mere absence of secondary granules. In these patients, neutrophil granules are atypically distributed in the cell and display an altered carbohydrate profile and protein composition. It remains to be determined in functional studies how the altered granule content impacts on microbe killing and if the absence of carbohydrate in granules influences cellular adhesive properties in SGD. Our lectin survey and proteomics comparison enabled a better characterization of SGD and raised hypotheses on the regulation of glycosylation and the control of nuclear shape during neutrophil maturation.

## Ethics Statement

This study was carried out in accordance with the recommendations of the institutional review boards of the Medical University of Vienna with written informed consent from all subjects. All subjects gave written informed consent in accordance with the Declaration of Helsinki. The protocol was approved by the institutional review boards of the Medical University of Vienna.

## Author Contributions

NS, JH, RD, EM, WG, BH, and AJ planned and performed all experimental work and interpreted the results. NS, JH, RD, and KB wrote the initial draft and finalized version of the manuscript with input from the other coauthors. Serial routine immunological characterization was performed by EM and AJ. JL and OZ provided clinical care and critically reviewed clinical and immunological patient data. KLB provided help for mass spectrometric experiments and data analysis. RK and DK provided laboratory resources and critically reviewed experimental data. KB conceived this study, provided laboratory resources, and critically reviewed and interpreted experimental and clinical data. All the authors agreed to the publication of this manuscript.

## Conflict of Interest Statement

The authors declare that the research was conducted in the absence of any commercial or financial relationships that could be construed as a potential conflict of interest.

## Abbreviations

BPI, bactericidal permeability-increasing protein; b-zip, basic leucine zipper; CEBPE, CCAAT/enhancer-binding protein-ε; ExAC, Exome Aggregation Consortium; HC, healthy control; HNP, human neutrophil peptide; LINC, linker of nucleoskeleton and cytoskeleton; MDS, myelodysplastic syndrome; MPO, myeloperoxidase; PMN, polymorphonuclear; PNA, peanut agglutinin; RBC, red blood cell; SGD, specific granule deficiency; UEA-I, *Ulex europaeus* agglutinin I.
