# Supplementary material for: CEBPE-Mutant Specific Granule Deficiency Correlates With Aberrant Granule Organization and Substantial Proteome Alterations in Neutrophils
Source: Front Immunol. 2018 Mar 29;9:588. doi: 10.3389/fimmu.2018.00588 (PMC5884887; doi:10.3389/fimmu.2018.00588)
Supplement: Supplementary file 1 [file image_1.PDF]

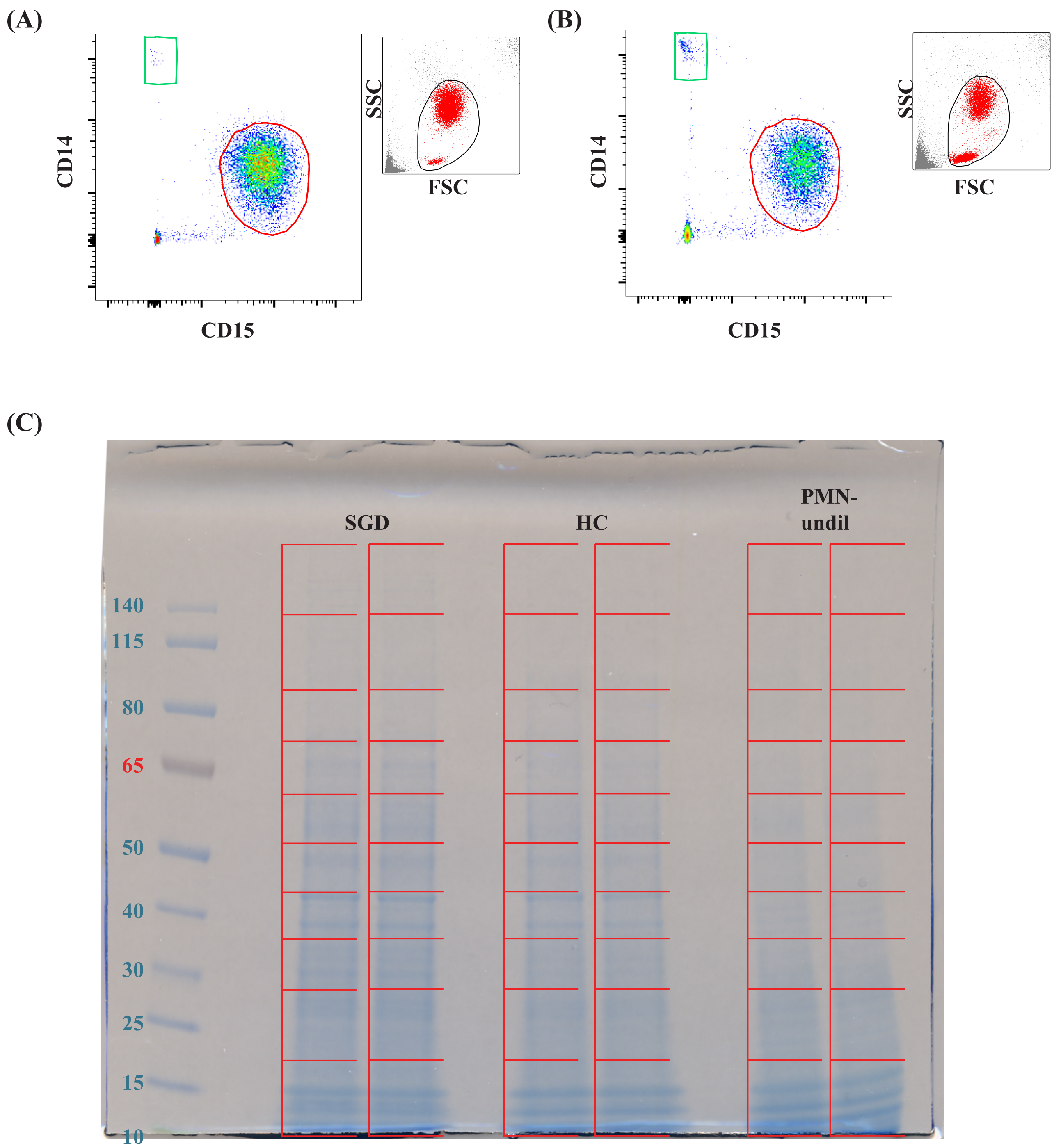

Supplemental Figure 1 - (A-B) Neutrophils isolated with normal (A) and reduced density Ficoll (B, see Khanna-Gupta 2007) from healthy controls. Neutrophil pellet was stained for CD14 and CD15. (C) Lysed neutrophil pellets of patient 1 (SGD) and a healthy control (HC) obtained with modified ficoll density, and a polymorphonuclear cell-enriched fraction of a healthy control (PMN-undil) were loaded on a SDS PAGE gel and sliced into 10 pieces followed by subsequent tryptic digest and mass spectrometric analysis.
