# Supplementary figures and images for: CEBPE-Mutant Specific Granule Deficiency Correlates With Aberrant Granule Organization and Substantial Proteome Alterations in Neutrophils
Source: Front Immunol. 2018 Mar 29;9:588. doi: 10.3389/fimmu.2018.00588 (PMC5884887; doi:10.3389/fimmu.2018.00588)

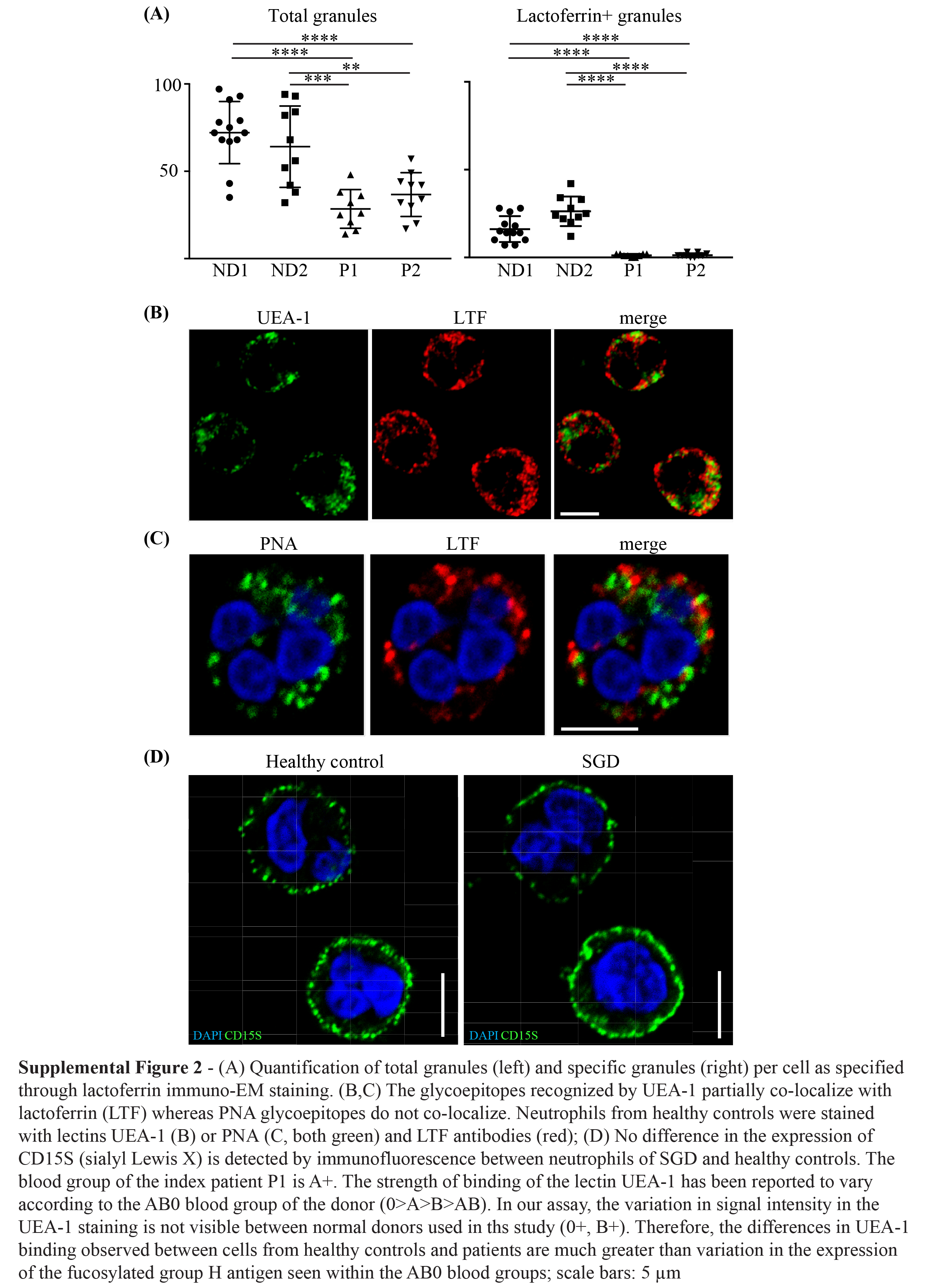

Supplement: Supplementary file 2 [file image_2.tif]
