# Supplementary material for: CEBPE-Mutant Specific Granule Deficiency Correlates With Aberrant Granule Organization and Substantial Proteome Alterations in Neutrophils
Source: Front Immunol. 2018 Mar 29;9:588. doi: 10.3389/fimmu.2018.00588 (PMC5884887; doi:10.3389/fimmu.2018.00588)
Supplement: Supplementary file 3 [file image_3.PDF]

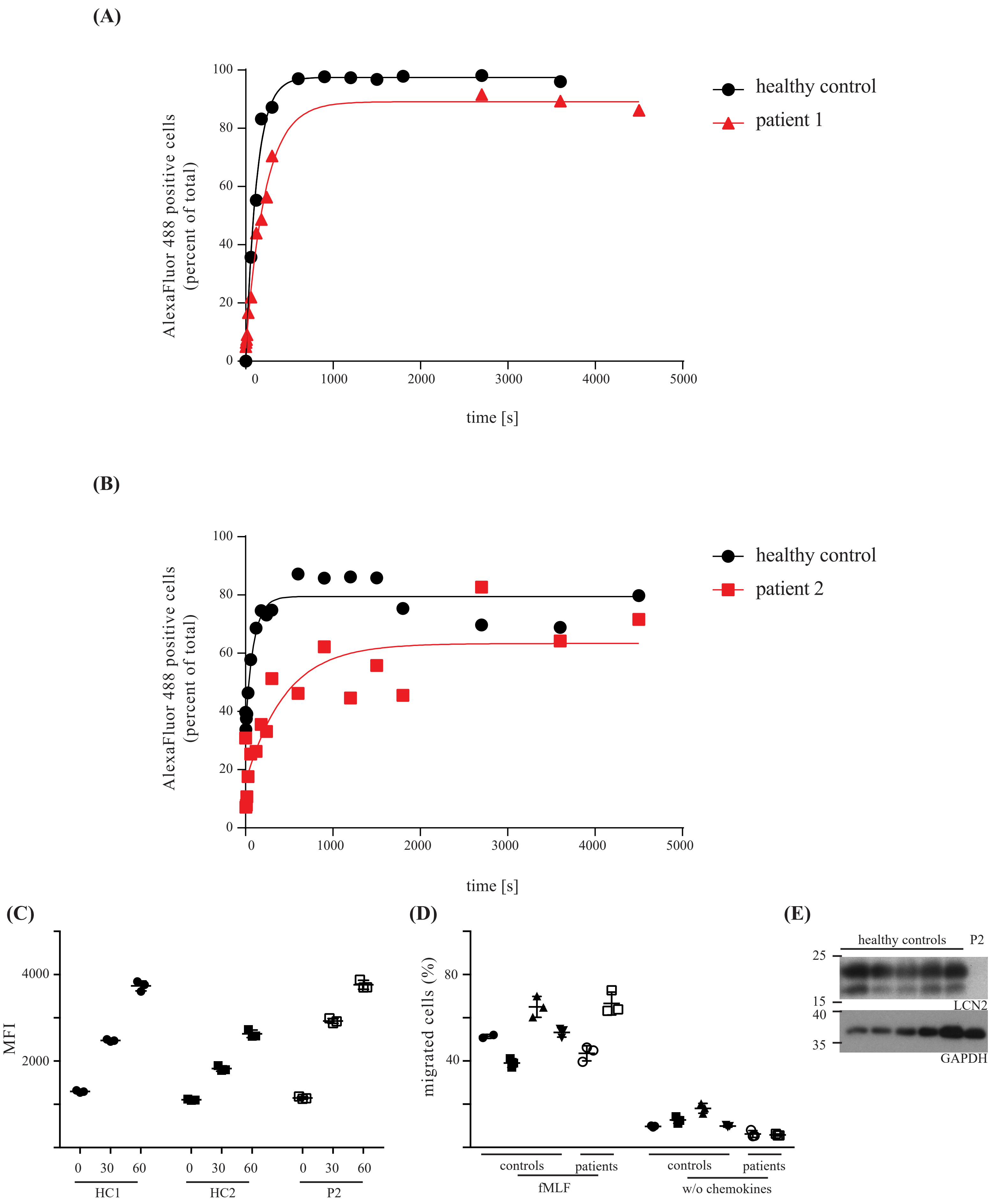

Supplemental Figure 3 - Neutrophil pellets of patient 1 (A) or patient 2 (B) and healthy controls obtained with modified ficoll density (see reference Khanna-Gupta 2007) were subjected to analysis of oxidative burst. (C). Phagocytosis assay of heat-inactivated *S.aureus* (MOI 50) after 0, 30 and 60 minutes. (D). Chemotaxis assay upon fMLF stimulation. (E). Western Blot for LCN2.
